# Geroscience infrastructure building in China

**DOI:** 10.1002/agm2.12085

**Published:** 2019-10-10

**Authors:** Xiang Liu, Gairong Huang, Baiyu Zhou, Pulin Yu

**Affiliations:** ^1^ Department of Geriatric Medicine Henan Provincial People's Hospital Zhengzhou China; ^2^ National Center of Gerontology Beijing Hospital Beijing China

**Keywords:** aging research, chronic diseases, geriatric medicine, geroscience, population aging

## 1 INTRODUCTION

Population aging poses a serious challenge on a broad spectrum of issues for both developing and developed countries. For people in the fields of aging research and geriatric medicine, the critical tasks include elucidating the relationship between aging and disease development and finding models of care for the elderly that transcend conventional clinical practices and health‐care service delivery systems intended for the general population. It is now recognized that distinctions should be made between processes accompanying aging that may be adaptive and those that contribute to increased disease risks.^1^ Also, as comorbidities are very common in the elderly and often share underlying pathological processes, the approach to managing individual diseases separately needs to be reconsidered. All this relies on the integration of research findings on aging with understanding disease processes and how it can be translated into preventive and therapeutic strategies as well as clinical practice. That is where geroscience comes in.

Aging biology research has generated a great deal of knowledge about cellular and molecular features and mechanisms underpinning aging. Meanwhile, geriatric medicine has evolved largely as a subspecialty of internal medicine, with a focus on care for the elderly but without much consideration given to how the aged body is different from its younger counterpart.^2^ Geroscience strives to bridge the gap between the two fields and facilitate the application of insights gained from basic biology aging research in the prevention and management of diseases commonly seen in the elderly. Geroscience is a relatively new concept, even more so in China. Its acceptance requires interdisciplinary team‐building and institutional reorganization, which has yet to happen in China. Therefore, when we talk about geroscience infrastructure building in China, it is not that conscious efforts have been made toward that end, but rather how progress in geriatric medicine and aging research achieved in recent years has the potential to be channeled into a geroscience‐oriented pursuit towards better understanding of mechanisms of aging and its contribution to age‐related chronic conditions in the future.

## 2 AN AGING POPULATION AND SENIOR HEALTH‐CARE CHALLENGES IN CHINA

The past four decades have witnessed impressive economic growth and drastic social transformation in China. Elevated living standards enable people to live longer and healthier lives. Life expectancies in the wealthier regions are approaching or have already surpassed those in economically highly developed countries. Similar to what has been experienced in other countries, major causes of death have shifted from infant mortality and communicable diseases to cardiovascular diseases and malignant neoplasms.^3^ Coupled with the recently ended one‐child policy, the country is now exhibiting some unique demographic characteristics that have no parallel in countries with comparable economic development levels.

According to the latest report by the National Bureau of Statistics, the population of China reached 1.39 billion at the end of 2018.^4^ The sixth national census showed that China's population in 2010 was 1.34 billion.^5^ These numbers are indicative of a dramatic slowdown in population growth and the overall population is widely expected to decline in the near future. Older adults, currently defined as those aged 60 years or above, numbered about 241 million at the end of 2018, accounting for 17.3% of the population.^4^ The number of people aged 65 or above stood at 158 million, or 11.4% of the population.^4^ According to estimates, the elderly aged 65 and over will reach 25% of the general population sometime between 2040 and 2050.^6^ As health literacy continues to improve and healthy aging becomes the norm, such an enormous elderly population will undoubtedly impose increasing pressures on an already inadequate health‐care system.

The question of how to cope with an aging population under relatively underdeveloped economic conditions was raised in the early 1990s. The government took notice and passed legislation and instituted programs to provide financial support and care for senior citizens, such as pension plans and health‐care access. More recently, a series of initiatives to improve health‐care coverage was launched and basic health insurance was expanded to cover close to the entire population, although benefits are still limited for the elderly in rural areas. However, serious problems concerning health‐care benefits remain outstanding. There are marked discrepancies between urban and rural areas over issues such as health‐care resources, health outcomes, and prevalence of diseases. Also, although substantial improvement in life expectancy and many other health‐related measures has been made across the country, the health‐care chasm persists between regions at different economic development levels. The eastern regions, especially the urban centers, for example, enjoy excellent health‐care facilities and well‐trained medical professionals, while some hospitals and clinics in the central and western regions are still struggling to provide even the most basic services for their residents.

In addition, there is an urgent need to overhaul the existing health‐care infrastructure and service delivery mechanisms in order to provide quality care for the elderly. Currently, most people do not have access to well‐equipped geriatric wards at major hospitals. Almost all nursing homes are suffering from a severe shortage of modern equipment and well‐trained personnel. These inequalities and deficiencies are unlikely to be adequately addressed anytime soon. Although efforts need to be made to expand and improve medical and senior care facilities, long‐term solutions would preferably come from strategies that can help prevent or slow down age‐related illnesses, which in turn would depend on a clear understanding of processes of aging and risk factors that predispose individuals to disease development so that effective intervention can be attempted and greater health span can be achieved.

## 3 GERIATRIC MEDICINE AND AGING RESEARCH IN CHINA

The establishment of geriatric medicine as a medical specialty in China was preceded by research programs led by several groups of physicians who sought to investigate factors contributing to longevity and senior‐care‐related issues in the 1950s, even though the country during that period had a very young population.^7^ The Chinese Medical Association (CMA) convened its first national conference on aging and geriatric medicine in 1964.^7^ At the conference, participants submitted proposals for the development of geriatric medicine and implementation strategies. After a hiatus of more than 10 years, the field started to take shape, first with the formation of geriatric organizations and research institutions at the provincial level, followed by the establishment of the Geriatric Medicine Branch under the CMA in 1981. As the demand for specialized care increased, independent geriatric departments started to emerge at large hospitals in major cities in the late 1980s and early 1990s. However, the majority of geriatric departments have been created since 2000. Now the field consists of a vast national network of geriatric care units, some of which have hundreds of beds and state‐of‐the‐art research laboratories.

The CMA Geriatric Medicine Branch is represented by prominent experts from regions across the country and manages activities that serve to improve knowledge and skills for practitioners in the field. Two major national conferences are held each year, attracting thousands of participants. To improve geriatric care quality, expert panels and committees of the CMA Geriatric Medicine Branch have issued numerous care protocols, standards, and guidelines. There are also dozens of credit‐awarding regular training programs based at major continuing education centers designed to update professionals on new knowledge and practice trends. In the past 20 years, research by the geriatrics community in China has also flourished and the subjects have been primarily concerned with new techniques and methods for the diagnosis and management of chronic conditions, such as cardiovascular disease, diabetes, Alzheimer's disease, and osteoporosis, which have become the major threats affecting the health and quality of life of the elderly. Despite the progress, there are still shortcomings. As a young medical subspecialty, many of its practitioners have come from another medical subspecialty. Consequently, single disease‐based management has become firmly entrenched. Important tools, such as comprehensive geriatric assessment, have not been incorporated into regular practice. This in part also reflects a lack of quality research offering a clear account of common pathways underlying chronic diseases or strong evidence for integrated therapeutic approaches.

Research on aging biology has also come a long way. As in many other countries, there has historically been a disconnect between aging biology research and geriatric medicine in China. Although some hospitals have research projects focusing on aging biology, most researchers in the field are from units related to biological sciences at universities and institutions affiliated with the Chinese Academy of Sciences. Funding for aging research in the past two decades has increased manifold and, as a result, more scientists are entering this field, but they still represent only a small minority in the life sciences. Researchers in aging biology used to work independently instead of forming groups and their interaction with clinicians was limited. Their research covers areas including cellular and molecular traits closely associated with aging, such as changes in gene expression, cellular structure, stem cell biology, and signal transduction.^8^, ^9^, ^10^ However, judged by the overall quality and significance of their work, most aging biology scientists in this country are still at the stage of trying to keep up with trends set by leading researchers abroad.

In recent years, Chinese universities and research institutions have been aggressively recruiting accomplished biologists with experiences in Europe and North America. Their arrival has already helped raise the country's profile in biomedical research, as evidenced by their highly seminal and influential achievements.^11^, ^12^ Some studies actually show signs of collaboration between researchers from different disciplines and very much represent what geroscience advocates, i.e, taking advantage of aging biology discoveries and technologies to promote understanding of aging and disease processes and characteristics and to create new treatment strategies. A research team recoded a single nucleotide within the *NRF2* gene in human mesenchymal stem cells, endowing enhanced self‐renew ability for these cells, which could potentially lead to therapies to slow down cellular senescence and delay functional decline.^13^ Another study revealed that natural variation of a neuropeptide, RGBA‐1, modulates the rate of aging in *Caenorhabditis elegans* through mediating glia‐neuron signaling.^14^ Systems biology approaches have also been used to build models with data from cellular and molecular studies to assess systematic aging and health.^15^ These examples clearly indicate that biologists in China have become an important force and are making significant contributions to our knowledge of health‐related aging processes.

## 4 INTEGRATION OF AGING RESEARCH WITH SENIOR HEALTH

Academic reorganization and reorientation in China have often been initiated by government agencies, which not only hold the purse strings, but also issue orders on the direction of research and the organizational framework that supports it. The enormity of aging‐related impact has prompted the government to commit unprecedented amounts of resources to aging research and geriatric medicine and to launch initiatives aimed at revamping the existing infrastructure in order to find innovative solutions to health problems of the elderly. Currently, most funding for research on aging and age‐related diseases is provided by a number of central government agencies, including the National Natural Science Foundation, the Ministry of Science and Technology (MoST), and the National Health Commission. Their provincial and municipal branches also have similar programs in place. The grant application process has been allegedly plagued by rampant favoritism and inefficiency.^16^ As countermeasures, new regulations have been introduced to close loopholes and even artificial intelligence tools will be used to eliminate bias and to speed up the process.^17^

More recently, the MoST has set up a special fund, earmarking it for a 5‐year program, named Active Health and Aging, to support projects ranging from mechanisms and patterns of aging to development of products for chronic disease management. The stated goal of the program is to preemptively identify risk factors and intervene early to promote senior health. There is an emphasis on comprehensive characterization, including the etiological and pathophysiological aspects of diseases and conditions—such as frailty and sarcopenia—that preferentially affect people of old age. Each of the projects will receive multimillion‐dollar funding and will be conducted at multiple centers. Given the scale of these projects, their successful implementation will generate valuable data that may add to or revise our understanding of these diseases and conditions.

To encourage further integration of basic aging research with age‐related diseases and to harness the power of scale, the National Health Commission and the MoST have made bold moves to alter the landscape of aging research and geriatric care in the country. In 2015, the National Health Commission approved the founding of the National Center for Geriatric Medicine and Gerontology. The center, based at Beijing Hospital, is tasked with a long list of responsibilities, including basic and translational aging research, clinical care, rehabilitation, training, and public health research.^7^ Under planning is a new campus for the center that will occupy an area of approximately 50 hectares and have a 1000‐bed clinical division and a 1000‐bed rehabilitation division, in addition to research and education facilities. Its regional affiliates have been or are in the process of being established.

In 2016, the primary center of the MoST‐led National Centers for Geriatric Disease Research also opened at Beijing Hospital and is in charge of planning, development, and coordination of the network. The primary center will eventually be joined by 21 secondary centers located at the country's top hospitals, which will undertake tasks based on their respective strengths in the subdivisions of geriatric medicine. Forty‐one more hospitals will serve as the bases for implementation of research projects. According to experts involved in the development process, the rationale for building such a network is to promote the translation of basic research findings into clinical applications and to collect evidence for evaluation of clinical treatment. Some provinces have formed their own alliances intended for clinical trials and epidemiological studies.

The advantages of such a top‐down approach were made abundantly clear by the speed at which these new institutions came onto the scene. It also shows the resolve of the government to find efficient ways to allocate scarce resources to advance the country's capabilities to tackle the health challenges of aging. After all, China's per capita spending on research and health care still lags far behind that of wealthier countries.^18^ However, these arrangements were largely driven by political will rather than achieved through merit‐based selection. This raises the question of whether the resources have fallen into the right hands. Aside from the issue of fairness, there is also the question of whether such extensive networks are too unwieldy to be effectively managed, especially considering that a clear chain of command does not yet exist.

## 5 A LOOK AHEAD

The essence of geroscience lies in that a clear understanding of aging processes will help identify common pathways and mechanisms contributing to the development of chronic diseases in the elderly, whereby means of intervention can be found. However, there is no tried‐and‐true model of how to transition from the present mode of research to a geroscience‐oriented approach. The geriatric care infrastructure in China has received notable upgrading and even more ambitious goals have been set. The emphasis on translational research and validation of clinical methods represents a huge step forward. Current approaches still show signs of heavy influence by the single‐disease‐management tradition, but the basic elements for the adoption of geroscience‐based practice are there. Increased communication and cooperation between Chinese researchers in aging and the international community will undoubtedly serve to expedite the transition process. Advances resulting from geroscience can not only lead to specific preventive and therapeutic tools to tackle common diseases of old age, but also help build up confidence for embracing population aging as an opportunity to engineer far‐reaching restructuring through which attitudes and policies reflect the recognition that the negative aspects of population aging can be minimized, while health span and, along with it, productivity and quality of life for seniors can be maximized.

## CONFLICTS OF INTEREST

Nothing to disclose.
